# Transcriptional control of fungal cell cycle and cellular events by Fkh2, a forkhead transcription factor in an insect pathogen

**DOI:** 10.1038/srep10108

**Published:** 2015-05-08

**Authors:** Juan-Juan Wang, Lei Qiu, Qing Cai, Sheng-Hua Ying, Ming-Guang Feng

**Affiliations:** 1 Institute of Microbiology, College of Life Sciences, Zhejiang University, Hangzhou, Zhejiang, 310058, People’s Republic of China; 2 School of bioengineering, Qilu University of Technology, Jinan, Shandong, 250353, People’s Republic of China

## Abstract

Transcriptional control of the cell cycle by forkhead (Fkh) transcription factors is likely associated with fungal adaptation to host and environment. Here we show that Fkh2, an ortholog of yeast Fkh1/2, orchestrates cell cycle and many cellular events of *Beauveria bassiana*, a filamentous fungal insect pathogen. Deletion of *Fkh2* in *B. bassiana* resulted in dramatic down-regulation of the cyclin-B gene cluster and hence altered cell cycle (longer G_2_/M and S, but shorter G_0_/G_1_, phases) in unicellular blastospores. Consequently, Δ*Fkh2* produced twice as many, but smaller, blastospores than wild-type under submerged conditions, and formed denser septa and shorter/broader cells in aberrantly branched hyphae. In these hyphae, clustered genes required for septation and conidiation were remarkedly up-regulated, followed by higher yield and slower germination of aerial conidia. Moreover, Δ*Fkh2* displayed attenuated virulence and decreased tolerance to chemical and environmental stresses, accompanied with altered transcripts and activities of phenotype-influencing proteins or enzymes. All the changes in Δ*Fkh2* were restored by *Fkh2* complementation. All together, Fkh2-dependent transcriptional control is vital for the adaptation of *B*. *bassiana* to diverse habitats of host insects and hence contributes to its biological control potential against arthropod pests.

Eukaryotic cell cycle and growth are under the control of cyclin-dependent kinases, and such control may occur at the post-transcriptional level via modification of target proteins or at the transcriptional level^1^. Transcriptional cycling is critical for cell cycle dependent activities, which require transcription factor (TF) complexes, such as SBF/MBF, Ace2/Swi5 and Mcm1/SSF, to activate expression of G_1_/S-, G_2_/M- and M/G_1_-dependent proteins^2^,^3^. Forkhead (FKH) proteins, known as winged helix proteins, are a conserved family of eukaryotic TFs, which share a typical region of ~100 amino acids spanning across a monomeric DNA-binding motif and hence are involved in many cellular processes, such as morphogenesis and cell cycle^4^,^5^. In higher eukaryotes, the forkhead box O family (FOXO) TFs play important role in antiaging and stress response activities^6^,^7^.

There are only four FKH proteins in *Saccharomyces cerevisiae*, comprising Hcm1, Fhl1, Fkh1 and Fkh2^8^. The latter two share a forkhead-associated domain (FHA), and hence are different from Hcm1 and Fhl1 and partially redundant in regulating periodic expression of cell-cycle-dependent genes in early M phase^9^,^10^. Fkh2 can bind cooperatively with Mcm1 for the induction of the cyclin-B gene cluster (CLB) regulatory elements in G_2_ phase^11^,^12^. The CLB2-cluster genes are usually transcribed in late S and G_2_ phases and also involved in G_2_/M transition^3^. These genes, such as *CLB2, CDC15, ACE2* and *SWI5*, control mitotic entry/exit and cytokinesis^13^,^14^. Transcriptional alteration of the CLB2-cluster genes by double deletion of *FKH1/2* has been shown to cause defects in nuclear migration, cell morphology, cell separation and aberrant cell cycle, and to alter gene expression towards pseudohyphal growth^10^,^12^,^15^. Fkh1 can support periodic transcription of the CLB2 cluster in the absence of Fkh2 and also participate in mating-type switching^15^,^16^. Double deletion of *FKH1/2* in the budding yeast may impede normal lifespan and stress resistance, hinting to their involvement in H_2_O_2_-induced cell cycle arrest^17^,^18^. Hcm1 and Fhl1 is linked to the mitotic function of calmodulin and the activity of RNA polymerase III respectively, and also, Hcm1 is best known to induce expression of G_1_/S genes, including Fkh1 and Fkh2, in late S phase^19^,^20^. *Schizosaccharomyces pombe* also harbors four FKH proteins, i.e., Fhl1, Sep1, Mei4 and Fkh2 which align with Fhl1, Hcm1 , Fkh1 and Fkh2 in *S. cerevisiae*^21^,^22^,^23^. Of those, Fhl1 acts as a regulator of pre-ribosomal RNA (rRNA) processing in association with the proteins involved in ribosome assembly^24^. Sep1 is linked to the regulation of the cell cycle and the periodic expression of the genes in the M and G_1_ phases^25^. Mei4 is a meiosis-specific TF^26^,^27^. Fkh2 can mediate periodic expression of many genes involved in cell cycle, sexual differentiation and stress responses^22^,^23^, and is also responsible for the intron retention of gene transcripts during vegetative growth and pre-meiotic S phase^28^. In addition, CaFkh2, an ortholog of *S. cerevisiae* Fkh1/2 in *Candida albicans*, has proved essential for morphogenesis^29^.

Unlike the advances in the yeasts, the FKH TFs are functionally unexplored in filamentous fungi. The genome of *Beauveria bassiana*, a filamentous entomopathogen widely applied for biological control of arthropod pests^30^, harbors only a single FKH TF orthologous to both Fkh1 and Fkh2 in *S. cerevisiae*^31^. This FKH TF is named as Fkh2 due to its closer relationship with Fkh2 in *S. cerevisiae* and *S. pombe*. The infection of an insect host by *B. bassiana* starts from conidial adhesion to the insect cuticle, followed by penetration of germ tubes through the cuticle for entry into the host hemocoel, in which multicellular hyphae turn into yeast-like unicellular blastospores for rapid propagation by budding until the host dies from mycosis^30^. Upon the host death, blastospores turn back to hyphae, which must penetrate again through the cuticle for outhgrowth and conidiophore development so as to produce conidia on cadaver surface for survival, dispersion or new infecton cycle. However, it is unclear how *B. bassiana* Fkh2 is involved in the regulation of cell cycle and biological control potential dependent upon multistress tolerance and virulence. Thus, this study sought to elucidate the functions of Fkh2 in *B. bassiana* by multi-phenotypic, transcriptional and biochemical analyses of its deletion mutant versus parental wild-type and complementary rescued mutant. We found that, in *B. bassiana,* Fkh2 was required not only for cell cycle progression, hyphal septation, and cell size and density but for many more biological processes of the fungal entomopathogen than of the model yeasts, including carbon/nitrogen utilization, asexual development, host infection, and cellular responses to fungicides, osmotic agents, high temperature and UV-B irradiation.

## Results

### Identification of Fkh2 in *B. bassiana*

A search through *B. bassiana* genome^31^ with the queries of all FKH sequences of *S. cerevisiae* and *S. pombe* in NCBI database resulted in only one Fkh2 sequence (NCBI accession code: EJP62625). The identified Fkh2 is characteristic with an FHA domain for the binding of phosphoproteins^32^ and a forkhead DNA-binding domain (FH), and phylogenetically closer to Fkh2 than to Fkh1 in the two yeasts (Fig. S1A). Sequence alignment analysis revealed 40–56% identity between the two domains of the identified Fkh2 and those of Fkh1/2 in *S. cerevisiae* (Fig. S1B). In addition, the *B. bassiana* genome also harbors the forkhead-type Sep1, Mei4 and Fhl1 homologs, which fall into distinctive clades. The Fkh2-coding sequence (1894 bp) amplified from the wild-type strain *B. bassiana* ARSEF 2860 (designated as Bb2860 or WT hereafter) encodes a protein sequence of 630 amino acids with the molecular mass of 68.7 kDa.

### *Fkh2* deletion alters cell cycle, hyphal septation and cell size

*Fkh2* was deleted from Bb2860 via homologous recombinaton of its 5´and 3´ fragments separated by the *bar* marker and rescued via ectopic integration of a cassette containing the full-length *Fkh2* (with flanking regions, 4895 bp) and the *sur* marker into Δ*Fkh2*. The recombination events were verified by PCR and Southern blot analyses (Fig. S2).

**Figure 1:**
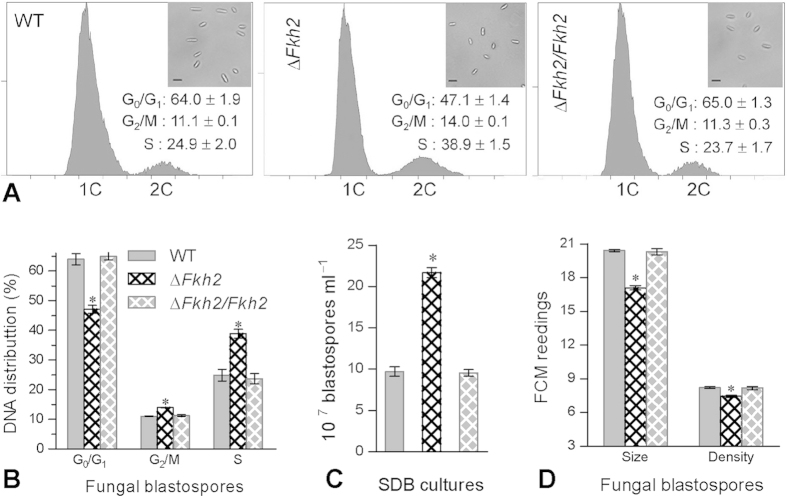
Deletion of *Fkh2* in *B. bassiana*alters cell cycle, size and density of unicellular blastospores. (**A**) Cell cycle (G_0_/G_1_, G_2_/M and S phases) of blastospores determined with DNA content profiles in FACS analysis and their microscopic images (scale bars: 5 μm). (**B**) Blastospore yields after 36 h incubation of 1 × 10^6^ conidia/ml SDB at 25 °C. (**C**) Blastospore size and density indicated by the FSc and SSc readings from the flow cytometry of 2 × 10^4^ cells per sample. The asterisked bar in each three-bar group differ significantly from two others unmarked (Tukey’s HSD, *P *< 0.05). Error bars: SD from three independent samples.

The cell cycle of unicellular blastospores in the Δ*Fkh2* culture differed from those in the control strains WT and Δ*Fkh2/Fkh2* (Fig. 1A). Fluorescence-activated cell sorter (FACS) analysis of 2 × 10^4^ blastospores stained with propidium iodide (a DNA-specific dye) revealed that Δ*Fkh2* had significantly longer G_2_/M and S, but shorter G_0_/G_1_, phases (Tukey’s HSD, *P *< 0.01) than WT (Fig. 1B). Consequently, Δ*Fkh2* produced 1.2-fold more blastospores than WT (Fig. 1C) after 36 h cultivation at 25 °C in Sabouraud dextrose broth (SDB). However, its blastospores suffered a mild, but significant, change in either size or density, as indicated by the readings of forward scatter (FSc) and side scatter (SSc) detectors from the flow cytometry of 2 × 10^4^ blastospores per sample (Fig. 1D).

**Figure 2:**
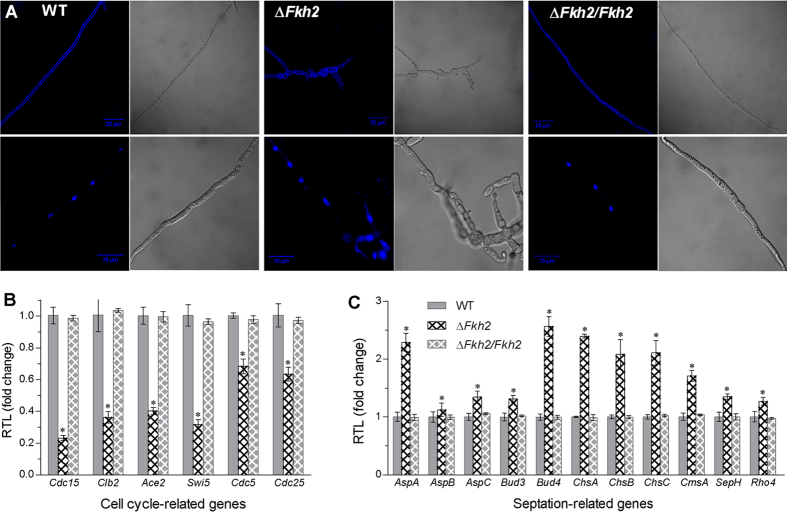
Deletion of *Fkh2* in *B. bassiana* affects cell cycle and septation pattern of multicullular hyphae and transcriptional profiles of related genes. (**A**) Bright and fluorescent images of hyphae stained with the cell-wall-specific dye calcofluor white (upper row) and the nucleic-acid-specific dye DAPI (lower row). Scale bars: 10 μm. (**B**,**C**) Relative transcript levels (RTL) of cell cycle and septation related genes in the 3-day-old SDB cultures of *Fkh2* mutants versus WT, respectively. The asterisked bar in each three-bar group differ significantly from two others unmarked (Tukey’s HSD, *P *< 0.05). Error bars: SD from three cDNA samples detected in qRT-PCR with paired primers (Table S2).

To reveal additional cell cycle changes, hyphae from 60 h SDB cultures were stained either with the cell-wall-specific dye calcofluor white or with the nucleic-acid-specific dye DAPI. As a result of bright/fluorescent microscopy, the stained Δ*Fkh2* hyphae showed denser septa and shorter/broader cells than those of the control strains (Fig. 2A), and ~20% of them became aberrantly branched (data not shown). These indicate that the mutant hyphae may have undergone abnormal mitosis.

In addition to the altered cell cycle and septation, four of six genes (*ACE2*, *CDC5*, *CDC15*, *CDC25*, *CLB2* and *SWI5*) required for the critical G_2_/M transition^33^,^34^,^35^ were down-regulated by more than 60% in Δ*Fkh2* (Fig. 2B). In contrast, five of 11 genes required for septum formation^36^ were remarkably up-regulated in Δ*Fkh2* relative to WT but unaffected in Δ*Fkh2/Fkh2* (Fig. 2C).

### *Fkh2* deletion increases cell sensitivity to nutritional stress

**Figure 3:**
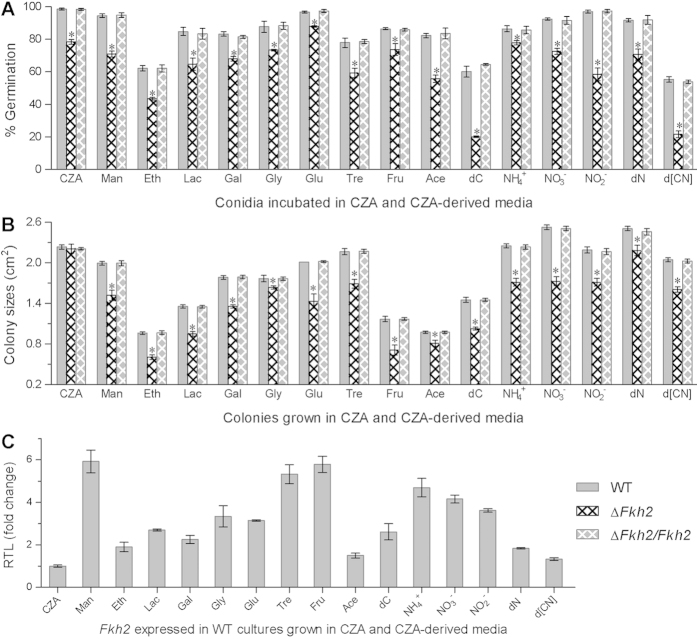
Deletion of *Fkh2* in *B. bassiana* alters cellular sensitivity to nutritional stress at 25 °C. (**A**,**B**) Percent germinations and colony sizes observed respectively after 24 h and 7 days of incubation on CZA and CZA-derived media with altered carbon/nitrogen sources and availability. Sole carbon source: mannose (Man), ethanol (Eth), lactose (Lac), galactose (Gal), glycerol (Gly), glucose (Glu), trehalose (Tre), fructose (Fru) or acetate (Ace). Sole nitrogen source: NH_4_^+^, NO_3_^–^ or NO_2_^–^. Availability: deletion of carbon (dC), nitrogen (dN) or both (d[CN]). The asterisked bar in each three-bar group differ significantly from two others unmarked (Tukey’s HSD, *P *< 0.05).(**C**) Relative transcript level (RTL) of *Fkh2* in the WT cultures grown on the CZA-derived media versus on CZA. Error bars: SD from three replicates (**A**,**B**) or cDNA samples (**C**).

Germination of the Δ*Fkh2* conidia was significantly lower than that of the WT conidia in minimal Czapek agar (CZA) and 15 CZA-derived media with altered carbon/nitrogen source and availability (Fig. 3A). The use of acetate, mannose, sucrose and lactose as sole carbon source decreased the mutant germination (by 20–26%) more than of glucose, fructose, glycerol and galactose (by 9–15%) compared with an intermediate effect of ethanol and trehalose (by ~18%). Among the tested nitrogen sources, NO_2_^–^ reduced the mutant germination (by 40%) more than NH_4_^+^ (by 9%) and NO_3_^–^ (by 20%). The starvation of carbon, nitrogen and both reduced the mutant germination by 40%, 21% and 34%, respectively.

After 7 days of cultivation at 25 °C, all the Δ*Fkh2* colonies on 15 CZA-derived media were more or less smaller than those of two control strains (Fig. 3B). Remarkably, colony size of Δ*Fkh2* was reduced by 39% on the sole carbon source of fructose, followed by 29−37% reductions on the carbon sources of ethanol, lactose and glucose respectively. Its growth on the nitrogen sources of NO_3_^−^, NH_4_^+^ and NO_2_^−^ was slowed down by 22−32%. Removal of sucrose, NO_3_^−^ and both from CZA resulted in the mutant colonies decreased by 29%, 13% and 21%, respectively.

Transcriptional expression of *Fkh2* in the WT cultures grown in the CZA-derived media (relative to CZA) for 4 days at 25 °C was up-regulated by up to 4.8-fold for the tested carbon sources except acetate or ethanol and by 2.6- to 3.9-fold for the tested nitrogen sources (Fig. 3C). Taken together, Fkh2 was a positive regulator of the fungal response to the nutritional stresses of carbon/nitrogen starvation and different carbon/nitrogen sources during conidial germination and vegetative growth perhaps due to its transcriptional responses to the stresses.

### *Fkh2* deletion facilitates conidiation but reduces conidial viability, size and density

**Figure 4:**
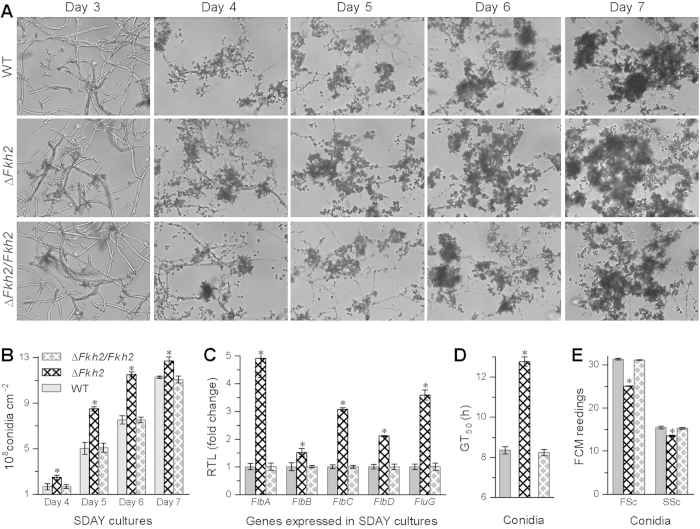
Deletion of *Fkh2* in *B. bassiana* facilitates conidiation, delays conidial germination and reduces conidial size and density. (**A**) Microscopic images of conidiating structures over the days of incubation on SDAY at 25 °C. (**B**) Conidial yields measured from the cultures. (**C**) Relative transcript levels (RTL) of conidiation-required genes in the SDAY cultures grown for 3 days. (**D**) Time length required for 50% conidial germination (GT_50_) on GM plates at 25 °C. (**E**) Conidial size and density indicated by the FSc and SSc readings from the flow cytometry of 2 × 10^4^ conidia per sample. The asterisked bar in each three-bar group differ significantly from two others unmarked (Tukey’s HSD, *P *< 0.05). Error bars: SD from three replicates (**B**,**D**) or three samples of cDNA (C) or conidia (**E**).

During 7 days of incubation at 25 °C in rich SDAY (Sabouraud dextrose agar plus 1% yeast extract), a standard medium for cultivation of entomopathogenic fungi, conidiophore development and conidiation occurred earlier in Δ*Fkh2* than in the control strains (Fig. 4A). Conidial yield in Δ*Fkh2* compared with WT increased by 49%, 69% and 53% on days 4, 5 and 6, respectively, although the yield increase diminished to only 13% on day 7 (Fig. 4B). The facilitated conidiation in Δ*Fkh2* was concurrent with remarked up-regulation (by 2.1*−*3.9 fold) of three transcription factors (FluG, FlbA and FlbC) required for conidiophore development and conidiation^37^,^38^ (Fig. 4C). However, the Δ*Fkh2* conidia took much (4.4 h) longer to achieve 50% germination under normal conditions (Fig. 4D) and also suffered 20% reduction in size and 13% reduction in density (Fig. 4E). All the changes were restored to WT levels by *Fkh2* complementation.

### *Fkh2* deletion increases cell sensitivities to oxidants, fungicides and osmotic agents

**Figure 5:**
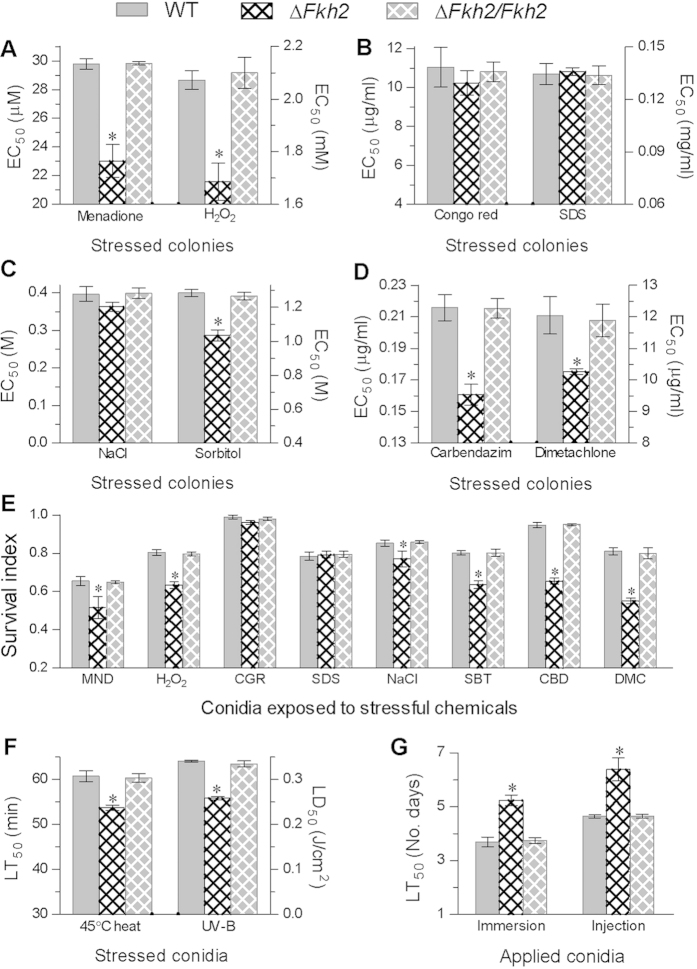
Deletion of *Fkh2* in *B*. *bassiana* reduces multistress tolerance and virulence. (**A**−**D**) Effective concentrations (EC_50_s) of different chemical stressors required to suppress 50% colony growth on CZA plates after 7 days of incubation at 25 °C. (**E**) Conidial survival index (relative germination) after 24 h incubation at 25 °C on GM plates containing a sensitive concentration of MND (menadione 0.2 mM), H_2_O_2_ (2 mM), CR (Congo red 0.5 mg/ml), SDS (0.3 mg/ml), NaCl (1.2 M), SBT (sorbitol 2 M), CBD (carbendazim 2 μg/ml) or DMC (dimetachlone 0.1 mg/ml). (**F**) LT_50_ (min) and LD_50_ (J/cm^2^) for conidial tolerance to 45 °C heat stress and UV-B irradiation, respectively. (**G**) LT_50_ (no. days) for the fungal virulence to *G. mellonella* larvae infected by topical application (immersion) of 10^7^ conidia/ml suspension or hemocoel injection of 500 conidia per larvae. The asterisked bar in each three-bar group differ significantly from two others unmarked (Tukey’s HSD, *P *< 0.05). Error bars: SD from three repeated assays.

The two control strains showed no significant difference in cellular sensitivity to the stress of oxidation (Fig. 5A), cell wall perturbation (Fig. 5B), high osmolarity (Fig. 5C) or fungicides (Fig. 5D) based on an effective concentration (EC_50_) of each chemical stressor required to suppress 50% colony growth during co-cultivation in CZA at 25 °C. Compared with the control strains, Δ*Fkh2* were significantly more sensitive (Tukey’s HSD, *P *< 0.05) to the oxidants menadione (23%) and H_2_O_2_ (19%), the fungicides carbendazim (25%) and dimetachlone (14%), and the osmotic agent sorbitol (19%) but showed no significant change in sensitivity to the osmotic salt NaCl and the cell wall stressor Congo red or sodium dodecyl sulfate (SDS).

Sensitivity changes also occurred in the Δ*Fkh2* conidia co-cultivated at 25 °C for 24 h with a sensitive concentration of each chemical stressor in a germination medium (GM). As indicated by conidial survival index (Fig. 5E) estimated as the ratio of percent germination in each stress treatment over that in an unstressed control, conidial germination in Δ*Fkh2* compared with WT was significantly suppressed by 21%, 21%, 31%, 32%, 21% and 10% in the treatments of menadione (0.2 mM), H_2_O_2_ (2 mM), carbendazim (2 μg/ml), dimetachlone (0.1 mg/ml), sorbitol (2 M) and NaCl (1.2 M), respectively, but insignificantly (Tukey’s HSD, *P *> 0.05) by either Congo red (0.5 mg/ml) or SDS (0.3 mg/ml). Apparently, Fhkh2 played more important role in the fungal responses to the oxidants and fungicides than to the osmotic agents but was not involved in the response to cell wall perturbation during conidial germination and vegetative growth.

### *Fkh2* deletion reduces biological control potential

As important traits of fungal biological control potential, conidial thermotolerance and UV-B resistance were significantly reduced in Δ*Fkh2* compared with the control strains, which exhibited similar LT_50_ (~61 min) and LD_50_ (~0.34 J/cm^2^) under 45 °C heat stress and UV-B irradiation (Fig. 5F). Analyses of the time-mortality trends of *Galleria mellonella* larvae infected by topical application of 10^7^ conidia/ml suspension and hemoceol injection of 500 conidia per larva resulted in the LT_50_s of 5.2 and 6.4 days for Δ*Fkh2* (Fig. 5G), respectively. The two estimates were significantly (1.5- and 1.7-day) longer than those from the control strains (~3.7 and ~4.7 days for the two types of bioassay respectively). Apparently, the fungal action to kill the larvae via the normal route of cuticular penetration was significantly delayed by the *Fkh*2 deletion (Tukey’s HSD, *P *< 0.05). In microscopic examination, however, hyphal bodies of the Δ*Fkh2* and control strains in the hemolymph samples, which were taken from the larvae surviving 3.5 days after injection, showed similar morphology and abundance (Fig. S3), hinting that the attenuated Δ*Fkh2* virulence through the cuticle-bypassing infection could be associated with the quality change of hyphal bodies as revealed in the *in vitro* blastospores.

### *Fkh2* deletion alters transcriptional profiles and activities of phenotype-associated proteins

**Figure 6:**
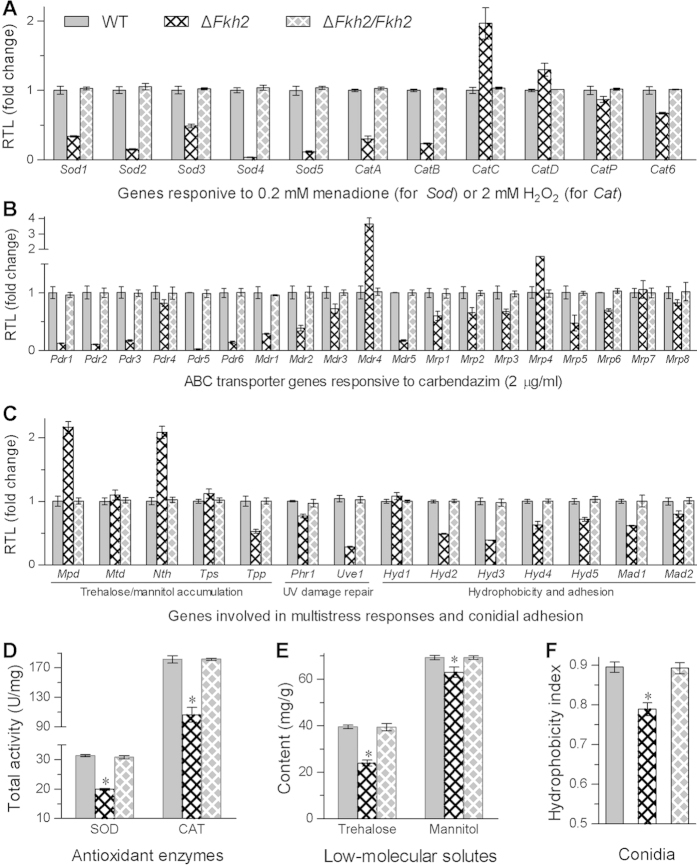
Deletion of *Fkh2* in *B. bassiana* alters transcriptional profiles and activities of phenotype-influencing proteins. (**A**−**C**) Relative transcript levels (RTL) of the genes associated with antioxidant reaction, multidrug resistance, trehalose/mannitol metabolism, UV damage repair and conidial hydrophobicity/adhesion, respectively. The qRT-PCR analyses with paired primers (Table S2) were run with the cDNAs derived from 4-day-old CZA cultures using the fungal 18S rRNA as an internal standard. (**D**) Total activities of SODs and CATs in the protein extracts from hyphal cells. (**E**) Intracellular trehalose and mannitol contents (mg/g dry mycelium mass) assessed in an HPLC system. (**E**) Index for conidial hydrophobicity determined with a diphase method. The asterisked bar in each three-bar group differ significantly from two others unmarked (Tukey’s HSD, *P *< 0.05). Error bars: SD from three independent samples.

To gain insight into the reductions of stress tolerance and virulence in Δ*Fkh2*, transcript levels of 44 genes associated with different phenotypes were assessed in cDNA samples derived from 3-day SDAY cultures of all the strains via quantitative real-time PCR (qRT-PCR). These genes were selected due to their known functions in *B. bassiana*, including those encoding superoxide dismutases (SODs)^39^, catalases (CATs)^40^, ATP-binding cassette (ABC) transporters^41^ and mannitol dehydrogenases^42^. Seven gene transcripts of 11 antioxidant enzymes were down-regulated by 51−96% in Δ*Fkh2* compared with WT (Fig. 6A), including all five SODs and two of six CATs. Similarly, ten of 19 examined ABC transporters associated with multidrug resistance were depressed by 53−98% in Δ*Fkh2* (Fig. 6B). Transcriptional changes of five enzymes involved in biosynthesis and degradation of trehalose and mannitol also occurred in the deletion mutant, accompanied with down-regulated transcripts of eight proteins involved in UV damage repair^43^, and conidial hydrophobicity and adhesion^44^ (Fig. 6C). Aside from these transcriptional changes, Δ*Fkh2* lost 36% and 41% of SOD and CAT activities in hyphal cells (Fig. 6D), 39% and 9% of intracellular trehalose and mannitol contents (Fig. 6E), and 11% of conidial hydrophobicity (Fig. 6F). All these changes were well restored by *Fkh2* complementation. These results indicated an importance of Fkh2 for the expression of phenotype-influencing proteins, supporting previous reports on the evolutionary conservation of Fkh1/2 that act at the *cis*-acting element to regulate functions in the budding yeast^45^,^46^.

## Discussion

As indicated by our data, Fkh2 is free of any paralogue in *B. bassiana* and can regulate transcriptional expression of many target proteins required for cell cycle, hyphal septation, asexual development, antioxidant reaction, multidrug resistance, UV damage repair and conidial adhesion, thereby exerting profound effects on the fungal cell cycle, morphogenesis, conidiation, cell size and density, carbon/nitrogen utilization, multistress tolerance and virulence. Our results indicate that the transcriptional control of Fkh2 is important for the fungal adaptation to host and environment, as discussed below.

First, deletion of *Fkh2* in *B. bassiana* resulted in remarkable down-regulation of the CLB2-cluster genes (*Clb2*, *Swi5*, *Ace2*, *Cdc5*, *Cdc15* and *Cdc25*) essential for the cell cycle and mitosis^33^,^34^,^35^, well in agreement with their transcriptional changes previously observed in yeast *Fkh1*/*2* double-deletion mutants^8^,^11^,^12^,^47^. The B-type cyclins, such as Cdc25, are activators of cyclin-dependent kinase 1 (Cdk1), whose suppression may result in an extension of G_2_/M transition^36^. Consequently, the cell cycle of yeast-like blastospores, which propagate by budding in insect hemolymph after fungal entry into the host homocoel, was altered in *B. bassiana*. As a global effect of the shortened G_0_/G_1_ transition and the elongated G_2_/M and S phases, the altered cell cycle resulted in a drastic increase of blastospore yield but a reduction in blastospore size or density. The alteration of the cell cycle in Δ*Fkh2* was also evident with more frequent formation of denser septa and shorter/broader cells in hyphae. The facilitated hyphal septation was supported by the up-regulated transcripts of several septation-required genes examined in this study, such as *AspA* required for septation^48^, *Bud4* essential for the selection of septation site by landmark proteins^49^, and *ChsA−C* involved in chitin synthesis at septation sites^50^,^51^,^52^.

Moreover, the altered cell cycle resulted in abnormal morphogenesis, i.e., faster conidiophore development and earlier conidiation, as observed in our Δ*Fkh2*. Consequently, its conidial yield was increased during normal cultivation. The facilitated conidiation was consistent with the fact that three of the examined transcripton factors essential for conidiophore development and conidiation^37^,^38^ were up-regulated in the 3-day-old SDAY culture of Δ*Fkh2*. However, aerial conidia produced by Δ*Fkh2* showed smaller size and lesser density like the submerged blastospores, and perhaps for thjs reason, their germination was much delayed. On the other hand, our Δ*Fkh2* mutant became more sensitive to nutritional stresses during conidial germination and colony growth but exhibited no morphological changes in either germ tubes or colonies under the stresses. Meanwhile, the *Fkh2* transcript was up-regulated by different degrees in the WT cultures grown under the nutritional stresses. These implicate that Fkh2 may take part in the use of environmental carbon and nitrogen sources by *B. bassiana*.

Furthermore, our Δ*Fkh2* mutant showed evolutionary conservation in the regulation of virulence and antioxidant activity, like attenuated virulence in *C. albicans* Δ*Fkh2*^29^ and decreased antioxidant activity in the deletion mutants of *S. cerevisiae* Fkh1/2^17,18^. However, the increased Δ*Fkh2* sensitivities to fungicides, high osmolarity, high temperature and UV-B irradiation were not revealed in previous studies. The increased Δ*Fkh2* sensitivity to oxidative stress is in agreement with a change of the same phenotype in the double deletion mutant of *S. cerevisiae Fkh1/2*, which has been shown to genetically interact with an anaphase-promoting complex in the yeast^17^. The alterations of multistress responses in Δ*Fkh2* are largely supported by the phenotype-affecting proteins changed at transcriptional and/or activity levels in this study. First, depressed transcripts of some SODs and CATs and their reduced activities were consistent with the Δ*Fkh2* hypersensitivity to the oxidative stress during conidial germination and radial growth. Despite multiple members in the fungal SOD and CAT families, only two MnSODs, i.e., cytosolic Sod2 and mitochondrial Sod3, and the catalases CatB and CatP were largely determinant to the total SOD and CAT activities in the hyphal cells of *B. bassiana*^39^,^40^, respectively. The expression of the mentioned enzyme genes except *CatP* was greatly depressed in our Δ*Fkh2*. Second, the increased sensitivities to two different fungicides were apparently attributable to the transcriptional depression of several examined ABC transporters associated with multidrug resistance in *B. bassiana*^41^. Third, the reductions of cellular osmotolerance, thermotolerance and UV-B resistance could be partially associated with the changed transcriptional profiles of five enzymes involved in the biosynthesis and degradation of trehalose and mannitol^42^ and hence the decreased accumulation of both low-molecule solutes in the mutant cells because the intracellular contents of such solutes are relevant to fungal multistress tolerance^53^,^54^. The reduced themotolerance in our Δ*Fkh2* was also associated with the down-regulation of *CatA*, which has been shown to contribute uniquely to conidial thermotolerance among the fungal CATs^40^. The decreased UV-B resistance was more likely attributable to not only a down-regulation of both *Uve1* and *Phr1* involved in UV damage repair^55^,^56^ but a decrease of intracellular SOD activity, which is positively correlated with the fungal UV resistance and virulence via increased defense response to host immunity upon entry into hemocoel^39^. Exceptionally, our Δ*Fkh2* mutant showed no change in sensitivity to the cell wall stressors Congo red and SDS during conidial germination and colony growth, hinting to little link of Fkh2 to cell wall integrity in *B. bassiana*. In addition, the suppressed transcripts of several genes required for conidial hydrophobicity and adhesion to host cuticle^43^,^44^ could be partially attributable to the attenuated virulence of Δ*Fkh2* via cuticular infection. However, microscopic examination of blastospores from the hemolymph samples of the injected larvae provided little evidence for its delayed kill action via cuticle-bypassing infection. We speculate that the delayed kill action could be likely associated with impaired blastospore quality, as revealed with smaller size and lesser density in the *in vitro* counterparts under submerged conditions.

In conclusion, Fkh2 participates in mediating the cell cycle and fundamental processes of *B. bassiana* at transcriptional level, such as hyphal septation, morphogenesis, conidiation, carbon/nitrogen utilization, multistress responses and host infection, in a way more subtle than the yeast homologs. Our findings highlight the significance of Fkh2 for the host and environmental adaptation and hence the biological control potential of *B. bassiana* against arthropod pests.

## Methods

### Microbial strains and culture conditions

The wild-type strain Bb2860 and its mutants were cultured at 25 °C on SDAY (4% glucose, 1% peptone, 1% yeast extract, 1.5% agar) for normal growth and on CZA (3% sucrose, 0.3% NaNO_3_, 0.1% K_2_HPO_4_, 0.05% KCl, 0.05% MgSO_4_ and 0.001% FeSO_4_ plus 1.5% agar) for phenotypic assays of fungal strains. *Escherichia coli* Top10 and *E. coli* DH5α from Invitrogen (Shanghai, China) were cultured in Luria-Bertani medium at 37 °C for plasmid propagation. *Agrobacterium tumefaciens* AGL-1 was incubated at 28 °C in the broth of 0.5% sucrose, 1% peptone, 0.1% yeast extract and 0.05% MgSO_4_) and used as a T-DNA donor for fungal transformation^57^.

### Structural and phylogenetic analyses of Fkh2 in *B. bassiana*

The FKH sequences of *S. cerevisiae* and *S. pombe* in NCBI protein database were used as queries to search for homologs in the Bb2860 genome under the NCBI accession NZ_ADAH00000000^31^ by BLAST analysis (http://blast.ncbi.nlm.nih.gov/blast.cgi). A single Fkh2 sequence found in the fungal genome was structurally compared with the yeast counterparts using online SMART program^58^, followed by phylogenetic analysis with MEGA5 software^59^.

### Generation of *Fkh2* mutants

First, the 5´and 3´ fragments (1427 and 1542 bp) of *Fkh2* were amplified from WT with paired primers (Table S1) using La*Taq* DNA polymerase from Promega (Madison, MI, USA) and inserted into the *Eco*RI/*Bam*HI and *Xba*I/*Spe*I sites of the backbone plasmid p0380-bar vectoring the phosphinothricin-resistant *bar* marker^39^, yielding the deletion plasmid p0380-5´Fkh2-bar-3´Fkh2. Second, the full-length coding sequence of *Fkh2* with flanking regions (4896 bp) was amplified from WT with paired primers (Table S1) and ligated into p0380-sur-gateway to exchange for the gateway fragment, resulting in the complementary plasmid p0380-sur-Fkh2 vectoring the sulfonylurea-resistant *sur* marker.

The method of *Agrobacterium*-mediated transformation^57^ was adopted to transform the deletion plasmid into WT via homogenous replacement of its partial coding sequence with the *bar* cassette and the complementary plasmid into Δ*Fkh2* via ectopic integration. Putative mutants grown on a selective medium were screened in terms of the *bar* resistance to phosphinothricin (200 μg/ml) or the *sur* resistance to chlorimuron ethyl (10 μg/ml). The recombination events were examined via PCR and Southern blot analyses with paired primers and an amplified probe of 422 bp (Table S1). For Southern blot analysis, all genomic DNAs extracted from the SDAY cultures were digested with *Eco*RV/*Nde*I. Positive Δ*Fkh2* and control strains were evaluated in triplicate experiments as follows.

### Examination of cell cycle and hyphal septation pattern

Aliquots of 50 ml conidial suspension in a nitrogen-limited broth (4% glucose, 0.4% NH_4_NO_3_, 0.3% KH_2_PO_4_ and 0.3% MgSO_4_) were incubated by shaking (110 rpm) at 25 °C for 4 days. Blastospores were collected from the liquid culture of each strain via filtration, washed twice with cold dd-H_2_O and fixed overnight in 70% ethanol. The fixed blastospores were resuspended in 15 ml of 50 mM sodium citrate (pH 7.5) supplemented with 1 μl RNase A (Sigma), followed by 30 min incubation at 37 °C to remove intracellular RNA. The suspension was then supplied with the DNA-specific stain propidium iodide (20 μg/ml). After 30 min staining at 4 °C, 500 μl aliquots of each stained suspension were subjected to FACS analysis in the flow cytometer FC500 MCL (Becman Coulter, CA, USA), yielding the readings of DNA concentrations. The G_0_/G_1_, G_2_/M and S phases of the cell cycle were determined based on unduplicated (1C), duplicated (2C), and intermediate DNA concentrations, respectively.

Hyphal septation and cell morphology were examined using the hyphae from SDB cultures shaken at 110 rpm for 2 days at 25 °C. Hyphae collected from the liquid culture were stained with calcofluor white and DAPI (Sigma) for 15 min, followed by visualization in the same bright/fluorescent field under a confocal microscope.

### Examination of morphogenesis, cell size and density

To assess conidiation capacity of each strain, the aliquots of 100 μl 10^7^ conidia/ml suspension were evenly spread on cellophane-attached SDAY (CO-SDAY) plates and incubated for 7 days at 25 °C in a 12:12 light:dark cycle. From day 3 onwards, three colony plugs (5 mm diameter) were bored daily from each plate using a cork borer and individually washed in 1 ml of 0.02% Tween 80. The concentration of conidia in the suspension was determined using a haemocytometer and converted to the number of conidia per cm^2^ plate culture. Daily culture samples were also examined under a microscope to reveal possible morphological changes between Δ*Fkh2* and control strains. Blastospore yields in the SDB cultures initiated with 1 × 10^6^ conidia/ml were quantified with microscopic counts after 36 h shaking at 25 °C.

Conidia and blastospores harvested from the SDAY and SDB cultures were suspended in PBS (pH 7.4). Three samples of each suspension were analyzed for cell size and density using the FSc and SSc readings from the flow cytometry of 2 × 10^4^ cells per sample. In addition, the time length (GT_50_) required for conidia to achieve 50% germination was assessed as a viability index of each strain by modeling analysis of percent germination trends over the time of incubation on GM plates (2% sucrose and 0.5% peptone plus 1.5% agar), which were spread with the aliquots of 100 μl conidial suspension and examined at 2 h intervals during 24 h incubation at 25 °C.

### Assessments of conidial germination and vegetative growth on different substrates

For each strain, conidial suspension was spread on the plates of CZA and 15 CZA-derived media with altered carbon/nitrogen source and availability. The derived CZA media were prepared by deleting 3% sucrose, 0.3% NaNO_3_ or both from the standard CZA, replacing the carbon source with 3% of maltose, ethanol, lactose, galactose, glycerol, glucose, trehalose, fructose or acetate (NaAc), and replacing the nitrogen source with 0.3% of NH_4_^+^, NO_3_^−^ or NO_2_^−^, respectively. After 24 h incubation at 25 °C, percent germination in each plate was estimated using three microscopic counts. Moreover, three aliquots of 1 μl 10^6^ conidia/ml suspension were centrally spotted on the plates of CZA and CZA-derived media, followed by 7 days of incubation at 25 °C and cross-measuring each colony diameter to compute its area (cm^2^) as an index of vegetative growth rate on each medium.

### Assays for cellular responses to chemical and environmental stresses

The aliquots of 1 μl suspension (10^6^ conidia/ml) were centrally spotted on the plates of CZA alone (control) or supplemented with a gradient of menadione (5−50 μM), H_2_O_2_ (1−4 mM), Congo red (2−100 μg/ml), SDS (50−300 μg/ml), NaCl (0.1−0.8 M), sorbitol (0.5−1.5 M), carbendazim (0.1−0.4 μg/ml) or dimetachlone (5−30 μg/ml). After 7 days of incubation at 25 °C, each colony diameter was cross-measured to compute colony area in each stress treatment and unstressed control. EC_50_ required for each chemical stressor to suppress 50% colony growth was estimated by modeling analysis of relative growth rates over its concentrations.

The effects of the chemical stresses on conidial germination of each strain were assayed by spreading 100 μl aliquots of conidial suspension onto GM plates containing menadione (0.2 mM), H_2_O_2_ (2 mM), Congo red (0.5 mg/ml), SDS (0.3 mg/ml), NaCl (1.2 M), sorbitol (2 M), carbendazim (2 μg/ml) or dimetachlone (0.1 mg/ml). After 24 h incubation at 25 °C, percent germination in each plate was determined using three microscopic counts, and the ratio of the percentage in each chemical treatment over that in the control was estimated as conidial survival index (i.e., relative germination) under the chemical stress.

Conidial thermotolerance and UV-B resistance were assayed by exposing conidial samples to 45 °C heat stress for up to 90 min and the irradiation of the weighted UV-B wavelength of 312 nm at the doses of 0.1−0.5 J/cm^2^, followed by modeling analysis of relative germination rates over the intensities of the stresses for LT_50_ (min) and LD_50_ (J/cm^2^) estimates of each strain, as described previously^60^.

### Bioassay for fungal virulence

The virulence of each strain against *G. mellonella* larvae (~300 mg *per capita*) from a vendor (Da Mai Chong Insectaries, Wuxi, Jiangsu, China) was bioassayed via two infection routes. Briefly, cohorts of ~30 larvae were immersed in 20 ml of 10^7^ conidia/ml suspension (treatment for cuticle infection) or 0.02% Tween 80 (control) for ~7 s, or inoculated by injecting 5 μl of 10^5^ conidia/ml suspension (treatment) or 0.02% Tween 80 (control) into the haemocoel of each larva. All treated cohorts were maintained in Petri dishes (15 cm diameter) at 25 °C for 7 days and monitored every 12 h for mortality. The sigmoid time-mortality trends attributed to different strains were differentiated with the LT_50_ estimates (no. days) made in probit analyses.

To observe the *in vivo* development of blastospores (hyphal bodies), hemolymph samples taken from five larvae surviving the injection of each strain for 3.5 days were dropped into 0.2 ml of anticoagulant solution^61^, followed by centrifugation and washing at 4 °C. The collected cells were resuspended in the solution for examination of their morphology under a microscope.

### Transcriptional profiling of *Fkh2* and its target genes

Total RNAs were extracted under the action of an RNAiso Plus Kit (Takara, Dalian, China) from the cultures of each strain grown at 25 °C in CO-SDAY for 3 days or in CZA alone or supplemented with menadione (0.2 mm), H_2_O_2_ (2 mm) or carbendazim (2 μg/ml) for 4 days, followed by reverse transcription into cDNAs under the action of a PrimeScriptH^RT^ reagent kit (Takara). The cDNA samples were used as templates to assess the transcripts of 49 candidate genes via qRT-PCR with paired primers (Tables S1 and S2) under the action of SYBR^®^ Premix Ex Taq^TM^ (Takara) because they are functionally involved in conidiation, antioxidation, multidrug resistance, trehalose/mannitol metabolism, UV damage repair, and conidial hydrophobicity and adhesion. The fungal 18S rRNA was used as an internal standard. The relative transcript level (RTL) of each gene was calculated as the ratio of its transcripts in the culture of Δ*Fkh2* or Δ*Fkh2/Fkh2* versus WT using the 2^−ΔΔCt^ method^62^. The same protocol was followed to assess the RTLs of six CLB2 cluster genes and 11 septation-required genes in each *Fkh2* mutant versus WT using cDNAs derived from 2-day SDB cultures and the transcriptional changes of *Fkh2* in the WT cultures grown for 3 days in CZA and CZA-derived media with altered carbon or nitrogen sources.

### Total activity assessments of SODs and CATs

Total SOD or CAT activity was quantified as described previously^39^,^40^. Briefly, protein extract from the hyphal cells of each strain was assayed for the SOD activity based on the inhibition of spontaneous autooxidation of pyrogallol (Sigma) as substrate and for the CAT activity by reading the spectrophotometric OD_240_ value as an index of of H_2_O_2_ decomposition. Protein concentration was assessed using a BCA (bicinchoninic acid) Protein Assay Kit (KeyGen, Nanjing, China). One unit of SOD activity in the protein extract was defined as the SOD amount required to inhibit 50% pyrogallol autoxidation rate while one unit of CAT activity was defined as 1 mM H_2_O_2_ consumed per min. The total SOD or CAT activity was expressed as U/mg protein extract.

### Assessments of intracellular mannitol and trehalose contents

Fungal masses (mycelia) were harvested from the 3-day-old CO-SDAY cultures, ground in liquid nitrogen, and resuspended in 2 ml dd-H_2_O. The suspension was boiled in a water bath for 1 h, followed by 30 min centrifugation at 16000 × *g*. The supernatant was assayed for the contents of mannitol and trehalose (mg/g dry mass) in an HPLC system using our previous protocol^54^.

### Assessment of conidial hydrophobicity

Conidia harvested from 7-day-old SDAY cultures were washed three times with a reaction buffer (160 mM K_2_HPO_4_, 52 mM KH_2_PO_4_, 30 mM urea and 1.7 mM MgSO_4_, pH 7.1), resuspended in the buffer and diluted to an optical density of ~0.4 at OD_470_ (*R*_1_). The aliquots of 3 ml each suspension were thoroughly mixed with 300 μl hexadecane by three cycles of 30 s vortex at 30 s interval. After standing for 15 min, the hexadecane phase was carefully removed from each tube, and the aqueous phase was kept for 30 min at 4 °C to remove the residual solidified hexadecane. Finally, the aqueous phase was transferred to room temperature for reading the OD_470_ value (*R*_2_). Conidial hydrophobicity of each strain was calculated as (*R*_1_−*R*_2_)/*R*_1_.

### Statistical analysis

All phenotypic observations, measurements and fitted estimates from the triplicate experiments were subjected to one-factor (strain) analysis of variance, followed by Tukey’s honestly significant difference (HSD) test to differentiate the means of each phenotype between Δ*Fkh2* and two control strains.

## Author Contributions

J.J.W. and M.G.F. designed the research. J.J.W. and M.G.F. analyzed the data. J.J.W., L.Q., Q.C. and S.H.Y. performed the experiments. M.G.F. and J.J.W. wrote the paper. All authors reviewed the manuscript.

## Additional Information

**How to cite this article**: Wang, J.-J. *et al*. Transcriptional control of fungal cell cycle and cellular events by Fkh2, a forkhead transcription factor in an insect pathogen. *Sci. Rep.* **5**, 10108; doi: 10.1038/srep10108 (2015).

## Supplementary Material

Supplementary Information
