# Supplementary material for: Transcriptional control of fungal cell cycle and cellular events by Fkh2, a forkhead transcription factor in an insect pathogen
Source: Sci Rep. 2015 May 8;5:10108. doi: 10.1038/srep10108 (PMC4424799; doi:10.1038/srep10108)
Supplement: Supplementary Information [file srep10108-s1.pdf]

Supplementary Material

Transcriptional control of fungal cell cycle and cellular events by Fkh2, a forkhead transcription factor in an insect pathogen

Juan-Juan Wang [1], Lei Qiu [1,2], Qing Cai [1], Sheng-Hua Ying [1], and Ming-Guang Feng [1]*

[1] Institute of Microbiology, College of Life Sciences, Zhejiang University, Hangzhou, Zhejiang, 310058, People's Republic of China (* Corresponding to M.-G. Feng. Email: mgfeng@zju.edu.cn)

[2] School of bioengineering, Qilu University of Technology, Jinan, Shandong, 250353, People's Republic of China

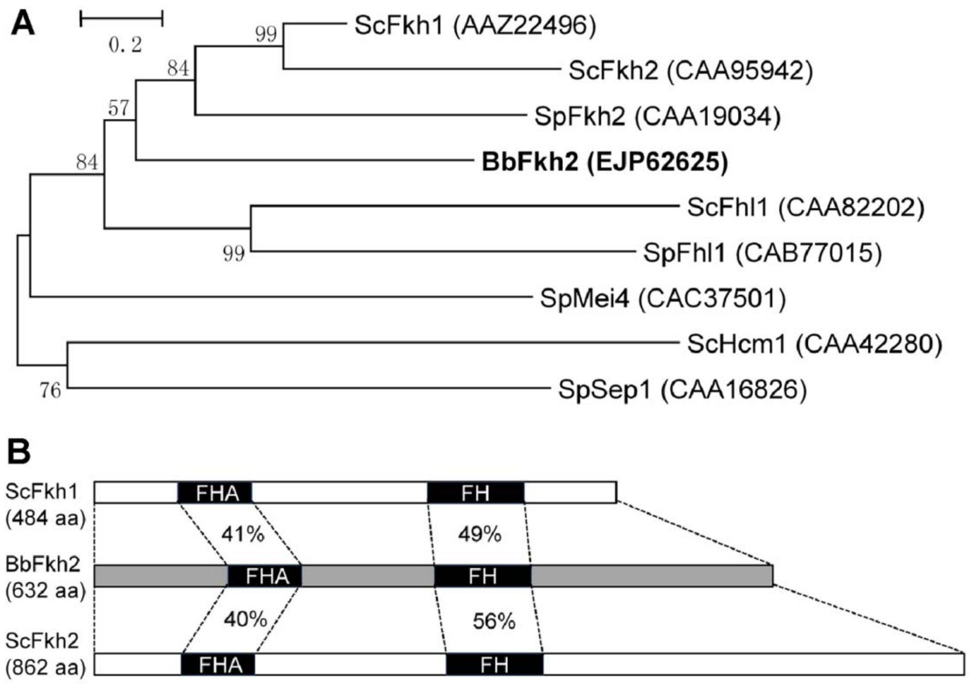

Figure S1 Features of Fkh2 in *B. bassiana* (Bb). (A) Phylogenetic tree constructed for comparison of BbFkh2 with the forkhead homologues of *S. cerevisiae* (Sc) and *S. pombe* (Sp) using neighbor-joining method in MEGA5 software. The bootstrap values of 1000 replications are given at nodes. (B) Protein sequence identity of BbFkh2 to ScFkh1 and ScFkh2.

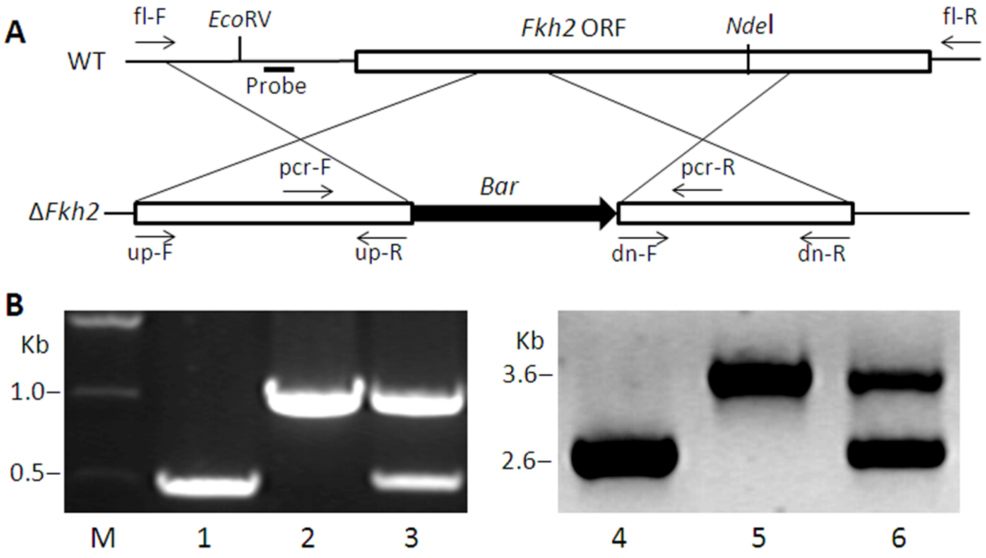

Figure S2 Constructing and identifying *B. bassiana Fkh2* mutants. (A) Schematic diagram for *Fkh2* deletion and complementation. (B) Detecting *Fkh2* in WT (lanes 1 and 4), Δ*Fkh2* (lanes 2 and 5) and Δ*Fkh2/Fkh2* (lanes 3 and 6) via PCR (lanes 1–3) with paired primers (Table S1) and Southern blot hybridization (lanes 4–6) with amplified probe. Genomic DNAs were digested by *Eco*RV/*Nde*I for the Southern blot analysis.

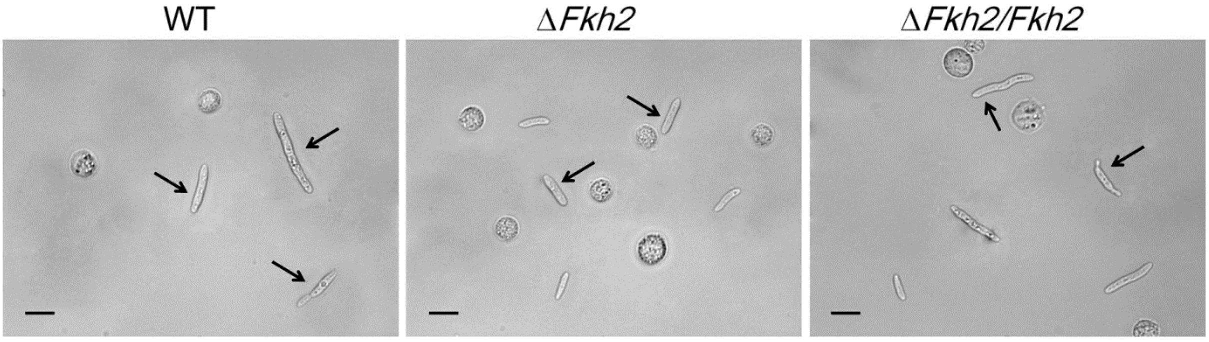

Figure S3 Microscopic images of blastospores (arrowed) in hemolymph of *G. mellonella* larvae surviving 3.5 days after intrahemocoel injection of 500 conidia per larva. Note that Δ*Fkh2* and two control strains showed little difference in blastospore morphology. Scale bars: µm.

Table S1 Paired primers used for the manipulation of *Fkh2* in *B. bassiana*.

Primers	Paired sequences (5'-3')*	Purpose
Fkh2up-F/R	AAAAAGAATTCTTGACAATGACAGAGGTGGC/AAAAAGGATCCGTGCAGCGCAGGTAGAGATA	Cloning *Fkh2* 5'-end (1427 bp)
Fkh2dn-F/R	AAAAATCTAGAAGAAGGCTACGTCGCCAGAG/AAAAAACTAGTGTGGCGTGCTTCCTATCTCG	Cloning *Fkh2* 3'-end (1542 bp)
Fkh2fl-F/R	ggggACCACTTTGTACAAGAAAGCTGGGTNGGTCACGGTCAACGAACA/ggggACAAGTTTGTAC AAAAAAGCAGGCTN TGGACAGTAACGAGGAAAGA	Cloning full-length *Fkh2* (4895 bp)
pFkh2-F/R	ACCTGGCACTCACTAAACGC/TGACTTGGGACACGATTTGG	PCR detecting *Fkh2*
sbFkh2-F/R	TTTTTGCGTATCAGAAGGACTC/CCTTGCTCTAAGTTTGCTGCTA	Southern blotting *Fkh2* (422 bp)
qFkh2-F/R	CTTGAACGGAATCTATACATAT/ATTGTGTCTGATGGAGTT	qRT-PCR detecting *Fkh2*
q18S-F/R	TGGTTTCTAGGACCGCCGTAA/CCTTGGCAAATGCTTTCGC	qRT-PCR detecting 18s RNA

* Underlined regions denote the sites of restriction enzyme for the disruption of *Fkh2* (*Eco*RI/*Bam*HI and *Xba*I/*Spe*I) or the fragments of gateway exchange for *Fkh2* complementation.

Table S2 Paired primers used for assessing transcript levels of phenotype-related genes via qRT-PCR.

Gene	Tag loci*	Annotation	Sequences of paired primers (5′-3′)
**Involved in conidiation**			
FbA	BBA_02968	Regulator of G protein signaling	CCAATCCACTCGCCGCTCTC/CGGAGGAAAGAGAATCGGTAGAGG
FlbB	BBA_06988	BZIP-type transcription factor	GCACTGACACGCCGACAAGAGC/CCGCCGCCGAAGCCTGTTG
FlbC	BBA_03181	Putative zinc finger protein C	TCCATCTCCAACTTGCTGGGTCTC/GGCGGCGTAGGCGGAAG G
FlbD	BBA_07259	Conidiophore development protein	CGGCAAGCGATGGGCAGAGATTG/ACGAGCAAGGTGACGGTAGAGGTG
FlbG	BBA_04942	FluG protein	CCTCCCTAGTTTGGTCGCTTTCTC/CGCTGTCGGTAATCTGCTCCTC
**Involved in G_2/M transition**			
Cdc15	BBA_02855	Cell division control protein	AGAGGCACTGGACAAGAG/GAACTGAGCGACGGAATAG
Clb2	BBA_08067	Cyclin domain-containing protein	CTTACCGAGATTAGCCTTC/CAGTTCTTCCTCAGTGTAG
Cce2	BBA_04604	C2H2 transcription factor	CGCAAGTCGGATCTTCAG/GGTGTGAGTTCGGATGTG
Swi5	BBA_01304	C2H2 transcription factor	GCCAGGTTCTCCGATAGC/CTGACGCATCTCTTGGAATAC
Cdc5	BBA_00468	Pre-mRNA-splicing factor	CGACCAGACACCATTGAC/CGTGACCTTGATATTGATACC
Cdc25	BBA_01974	Dual specificity phosphatase	GACGACCCTACTGACACCATAC/CTTGAAGAGTTGAGAAGCGAGAAG
**Involved in septin synthesis**			
ApsA	BBA_05547	Cell division/GTP binding protein	CTCTGCTCTGCTTCATAG/TTCTCGGTACGGTAGTTC
ApsB	BBA_06894	Cell division/GTP binding protein	TTACCGACAACATTGAAC/GGATGAAGAAGACACAAG
ApsC	BBA_04748	Cell division control protein 12	GGAGAAGTTCTTCAAGGT/AGGAGTCACGATTGTTAA
**Involved in chitin synthesis**			
ChsA	BBA_03236	Class 2 chitin synthesis	GTGTCTTACTGATTGCTA/ATAATGATGGCGTAGATG
ChsB	BBA_04667	Chitin synthesis 3a	CTCTGCCAATGTCACCAAG/TCAATGTCTTCCTGCTCCTT

ChsC	BBA_03590	Chitin synthesis 1	CGATCTGAACCAGCACAA/ATGACGGAGACGATGAGT
CsmA	BBA_04667	Chitin synthesis 3a	CTCTGCCAATGTCACCAAG/TCAATGTCTTCCTGCTCCTT
**Involved in landmark proteins synthesis**			
Bud3	BBA_03175	RhoGEF domain-containing protein	GATAATCCTATAATACGACTC/GTAGACATCATTGACAAG
Bud4	BBA_01075	GTP binding protein	GCGTCATCATCACCAGAA /TAATCTCATCATCCTCATCATACA
Rho4	BBA_03155	Ras family protein	CACATACTCCAAGAAGATT/AAGACGGTAGGAACATAT
**Involved in septation H protein synthesis**			
SepH	BBA_03315	Cell division control protein	AACTGCCTCAACATCATC/TCATATACACGCCAACAAG
**Involved in antioxidation**			
Sod1	BBA_02311	Cytosolic Cu/ZnSOD	GCGGCTTCCACATCCACACCTTTG / GGTCCAGCGTTGCCAGTCTTGAG
Sod2	BBA_09706	Cytosolic MnSOD	CCAGTGTTTGGCATTGACATG / TCAGCCGTCTTCCAGTTGATG
Sod3	BBA_09382	Mitochondrial MnSOD	TCTCCGGCAAGATTATGGAGC / TTGGCGTCATTCTTGGCCT
Sod4	BBA_04317	Mitochondrial FeSOD	CGAGATGGTCCTTACGGCTTCAG / GCTCCCAGGTGTTGAGGCATAG
Sod5	BBA_01984	Cell wall-anchored Cu/ZnSOD	CGGCGACCTCAGCGGCAAGTAC / GCCAGCAACAACAGGGACCGTAGG
CatA	BBA_06186	Catalase A	CCGTCTGGGCATCAACTGGGAAG / GCTGGGCGTGGTCGTGGTAG
CatB	BBA_05603	Catalase B	CCTCTGACGTTGGCGGCCCTTTC / CCGTGTCCGTGCTGCCTCGTG
CatC	BBA_09109	Catalase C	GAGGAGCCCAGCAACGCACAAGAG / TGAGGACGACAAGGCCGCCATTC
CatD	BBA_09760	Catalase D	CGGCTGCGGTGTCTTGTCCATAC / CCTTGTCGGCGTTCTGGCGAAG
CatP	BBA_09338	Catalase P	GCTGGGCTGATCTGCTGGTCCTTG / TCCTTGCTGTAACGGTGGCTGTCG
Cat6	BBA_06567	Catalase-like protein	TCAAGTCGGTTCAGGAGATGGAG / TTGTTGCGTCTTCAATCGGAGTG
**Involved in multidrug resistance**			
Pdr1	BBA_01781	ABC transporter (Pdr1)	CACTGCTAGATTGCTTGGCTGACC / ACCGTCGTTGTCTGGAGATGGAG
Pdr2	BBA_08779	ABC transporter (Pdr2)	CGACGAGACGCAGGTTCATTCTTC / GACAGCCGAAGGAGCCAATGC
Pdr3	BBA_05015	ABC transporter (Pdr3)	GCGAGCCTGCCCAATTCTTTCTG / GCTTGTGAGACGGTCCTGGTAGAG
Pdr4	BBA_01103	ABC transporter (Pdr4)	GCCAGTTCATTGCCGCCTACG / AAGGAGACGAGGACACCGATGATG
Pdr5	BBA_07660	ABC transporter (Pdr5)	TCCTGCCCTTCTTCCTCGTCATG / AGAGCACGCCGCCGACATAG
Pdr6	BBA_02556	ABC transporter (Pdr6)	GCCTCACCGTCTTCTTCACCAATG / GCTGTGCCTGCTCCTCATCTTTG
Mdr1	BBA_01025	ABC transporter (Mdr1)	TGCTGCCTGTGCCGTCTCC / TGGACCGCCGCTGGAAGTG
Mdr2	BBA_08160	ABC transporter (Mdr2)	CACGCCGCCAAGGTCGCCAACTG/ CGCTGCTTCTGCCCGCCCGAGAG
Mdr3	BBA_04716	ABC transporter (Mdr3)	GCGGTCTTGTCGGTCCCTCTG / GCGGCACCACGGCAATGTC
Mdr4	BBA_06993	ABC transporter (Mdr4)	CGCTTCTACGACCCGACCTCTG / TGCTGGCACGCCGAGATAACC
Mdr5	BBA_03584	ABC transporter (Mdr5)	GCTCGCTGCCAACAAGGACAGTG / TCTTCGCCCTGCTCCCCTTCAATG
Mrp1	BBA_10584	ABC transporter (Mrp1)	TCTTGGTTGGGCATTCGGCTTGG / TTGCTGAGTATGGTCGCTGCTGTC
Mrp2	BBA_05780	ABC transporter (Mrp2)	GCAAGATTAACCTTCTCACAA / CATCATAACGAACAGTCACAT
Mrp3	BBA_06577	ABC transporter (Mrp3)	GAGCAGCAGCATTGTATC / GTGAGATGGCAAGTTGATG

Mrp4	BBA_07810	ABC transporter (Mrp4)	CGGCTTACACGCAGAGTT / GCTCGGCTTCTCCAATCAG
Mrp5	BBA_03577	ABC transporter (Mrp5)	GCCACCATAAGAGCATTC / ACCAACGACTGAAGATGTA
Mrp6	BBA_05210	ABC transporter (Mrp6)	TATGATAGCGTGGTGGTTAT / TTGAGAATCGTCTGGAGTT
Mrp7	BBA_08610	ABC transporter (Mrp7)	ATCAGCAACATCATCCAATC / AGCAGCCAATAGTTAGCA
Mrp8	BBA_03854	ABC transporter (Mrp8)	GAATGTTACCGTGCGATAC / CCAGATATTATGTGCGTGAAT
**Involved in multistress responses**			
Mpd	BBA_02141	Mannitol-1-phosphate dehydrogenase	AACAAAAACGCCGAGGAGTC / CGGAGACAACCTGGTTGACAA
Mtd	BBA_06629	Mannitol dehydrogenase	ACGCTCTGATAGGCTCCATCA / TGATGGCGCGGTAGTTTGTA
Nth	BBA_01127	Neutral trehalase	CATTGAGCACTACGGCAAGATTC / TGATGGCGGCAAGAATGGAG
Tps	BBA_01532	Trehalose-6-phosphate synthase	CGTGTGCCTGGTGTCTTCC / GACTGCCGTTGAGCGACTG
Tpp	BBA_00676	Trehalose-6-phosphate phosphorylase	TGGCTGGTAAGGCGAACATTG / AGGATTATGGTAACGGCTGATGAG
**Involved in UV damage repair**			
Phr1	BBA_01664	Photolyase	ACGAACGGAATCAAGAAG / TTGTGTTGCTCATCTCTG
Ue1	BBA_06710	UV-endonuclease	GGTTAGGCTATGCTTGTT / ATGCCGATGTTCTAGGAT
**Involved in conidial adhesion**			
Hyd1	BBA_03015	Hydrophobin	CAAGACTGGCGACATTTG / ACAAGCTGGTTGAGAAGA
Hyd2	BBA_06599	Hydrophobin	CGAGAAGACGACCAAGAT / GAGAAGGTGGGAAAGAAGA
Hyd3	BBA_00530	Hydrophobin	GATATTACAGGCGGCAAT / TGACCACCAGGAATAGAG
Hyd4	BBA_03071	Hydrophobin	CGTCTTTGCTTCTCTCAT / AATTCATCTGCGTTACGA
Hyd5	BBA_02999	Hydrophobin	ATGAAGTTCTTTGCTATCG / CAGACAAGGTTGGAGTAG
Mad1	BBA_02419	Adhesion protein Mad1	CTTGAAGATGTGGTTGGAT / CCTGGAAGAAGTTGATGG
Mad2	BBA_02379	Adhesion protein Mad2	ATGAAGTCCTTTGCCATTGC / AATCTCGCCGTCAGTGAG

* Gene accession codes in the annotated genome of *B. bassiana* under the NCBI accession NZ_ADAH00000000.
